# Copper and Silver Trispyrazolylborate-Phosphinoazide Complexes: Synthesis, Characterization, and Nitrene Generation

**DOI:** 10.1021/acs.inorgchem.4c04397

**Published:** 2025-01-02

**Authors:** Manuel R. Rodríguez, Francisco Molina, M. Mar Díaz-Requejo, Pedro J. Pérez

**Affiliations:** Laboratorio de Catálisis Homogénea, Unidad Asociada al CSIC, CIQSO-Centro de Investigación en Química Sostenible and Departamento de Química, Universidad de Huelva, 21007 Huelva, Spain

## Abstract

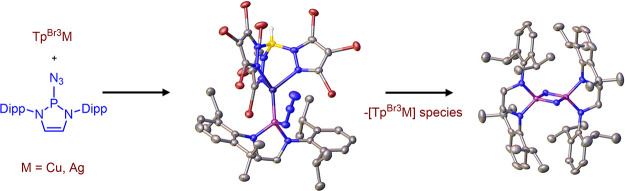

Phosphinoazide complexes of the composition Tp^Br3^M-L (M = Cu, Ag, and L = 2-azido-1,3-bis(2,6-diisopropylphenyl)-2,3-dihydro-1*H*-1,3,2-diazaphosphole) have been synthesized and structurally characterized. Their thermal decomposition led to cyclodiphosphazenes as a result of the metal-mediated coupling of two nitrene units in a process that takes place in both a stoichiometric and catalytic manner. Experimental data have allowed proposing a mechanistic pathway for this new transformation.

## Abstract 2

Phosphinoazide complexes of the composition Tp^Br3^ M-L (M = Cu, Ag, and L = 2-azido-1,3-bis(2,6-diisopropylphenyl)-2,3-dihydro-1*H*-1,3,2-diazaphosphole) were synthesized and structurally characterized, with a focus on their thermal decomposition pathway leading to the formation of cyclodiphosphazenes.

## Introduction

**Scheme 1:**
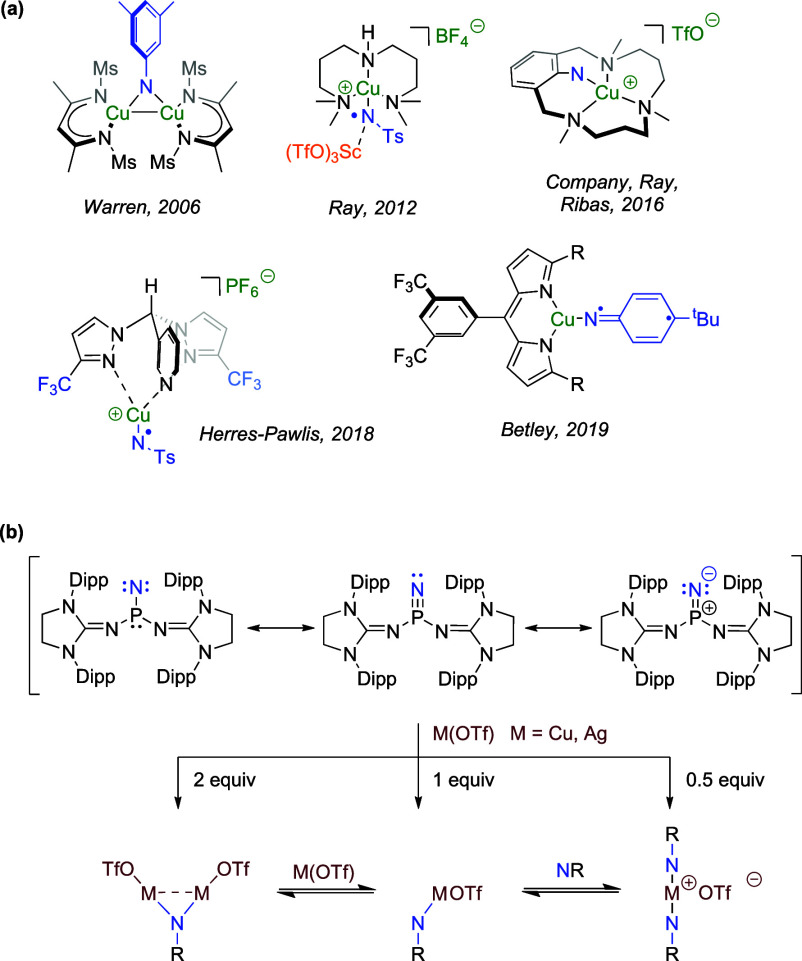
(a) Representative Examples of Detected or Isolated Copper-Nitrene Species Relevant to C–N Bond Formation; (b) Phosphinonitrenes Described by Bertrand and Its Coordination to Copper and Silver Centers

Metal-nitrene species are known as key intermediates in several chemical transformations involving the formation of C–N or N–heteroatom bonds.^1^ During the last few decades, great efforts have been directed toward the detection and isolation of these short-lived, highly reactive species, with the aim of developing a better understanding of their role in such reactions.^2^ Among the several metals employed in these catalytic transformations, copper and silver have frequently been reported with excellent results. Due to their high reactivity, few examples of isolated metal nitrenes are known for copper (Scheme 1a),^3^ whereas for silver, they are yet unknown.

One of the strategies that has been successfully applied for the stabilization and subsequent study of metal-nitrene complexes is the direct coordination of a highly stabilized free nitrene to a metallic center. This route requires that the nitrene is stable enough to allow its sequential generation and coordination to the metal, which has been proven to be possible only in the case of highly sterically demanding phosphinonitrenes. The first examples of this strategy were reported by Bertrand and Majoral, who irradiated a phosphinoazide in the presence of several trapping agents.^4^ Products derived from their addition across a hypothetical P≡N triple bond of the nitrene intermediate were detected. Furthermore, photolysis of the phosphinoazide in the absence of any other reagent resulted in the formation of a cyclodiphosphazene due to the dimerization of two transient phosphinonitrene units.^5,6^ Years later, the introduction of highly sterically demanding imidazolydiniminato groups bonded to the phosphorus atom allowed avoiding nitrene dimerization, leading to the isolation of the first free nitrene (Scheme 1b).^7^ Coordination of this nitridophosphane(V) to copper and silver centers was carried out using their triflic salts as precursors. However, the lack of steric protection favored the existence of an equilibrium between mono- and dinuclear species, precluding isolation of the former. More recently, two outstanding contributions regarding the isolation of free nitrenes have been published.^8^

Based on these foundations, we decided to merge our trispyrazolylborate-based copper and silver catalytic nitrene chemistry^9^ with phosphinoazide reagents aiming at inducing the formation of metal nitrenes from metal-phosphinoazide adducts. Herein, we report the synthesis, characterization, and reactivity of such complexes that undergo nitrogen extrusion and metal-nitrene formation as well as subsequent nitrene coupling reactions.

## Results and Discussion

### Synthesis of the Complexes Tp^Br3^M[P(N_3_)(DDD)] (M = Cu, 2; Ag, 3)

**Scheme 2:**
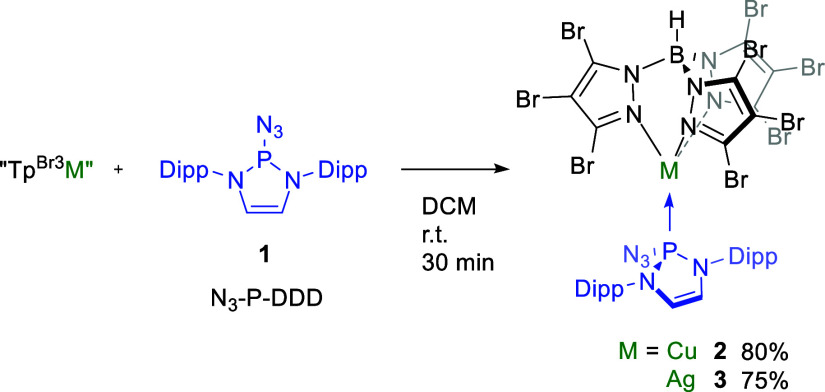
Synthesis of Tp^Br3^ M[P(N_3_)(DDD)] Adducts

We first targeted the synthesis of the corresponding adducts of the complexes bearing Tp^Br3^M cores (M = Cu, Ag) with the bulky phosphinoazide **1** (azido-1,3-bis(2,6-diisopropylphenyl)-2,3-dihydro-1*H*-1,3,2-diazaphosphole, N_3_–P-(DDD), Scheme 2). The addition of **1** onto solutions of Tp^Br3^Cu(NCMe) and [Tp^Br3^Ag]_2_ in DCM led, after 30 min of stirring and the corresponding workup, to the isolation of complexes Tp^Br3^M[P(N_3_)(DDD)] (M = Cu, **2**; Ag, **3**). Colorless crystalline materials of these new compounds were collected (yields = 75–80%) upon crystallization at room temperature, with some of the crystals being suitable for X-ray diffraction studies

Complexes **2** and **3** have been characterized by spectroscopic (^1^H, ^13^C, ^11^B, and ^31^P NMR) and analytical data. The spectra are deceptively simple, showing the expected resonances for Tp^Br3^ and the phosphine ligand. The ^31^P{^1^H] spectra for both complexes are quite different. A broad singlet centered at 108 ppm is found for the copper complex **2**, whereas two doublets centered at 116.9 ppm are observed for the silver analogue. These two resonances correspond to the coupling of the ^31^P nucleus with the two magnetically active nuclei of silver, ^107^Ag and ^109^Ag (δ 116.9, d, ^1^*J*_Ag(107)–P_ = 1030.2 Hz; 116.8, d, ^1^*J*_Ag(109)__–P_ = 888.8 Hz).^10^ As proof of the coordination of the ligand through the P donor, it is worth mentioning that free phosphine **1** appears at 121 ppm.

**Figure 1:**
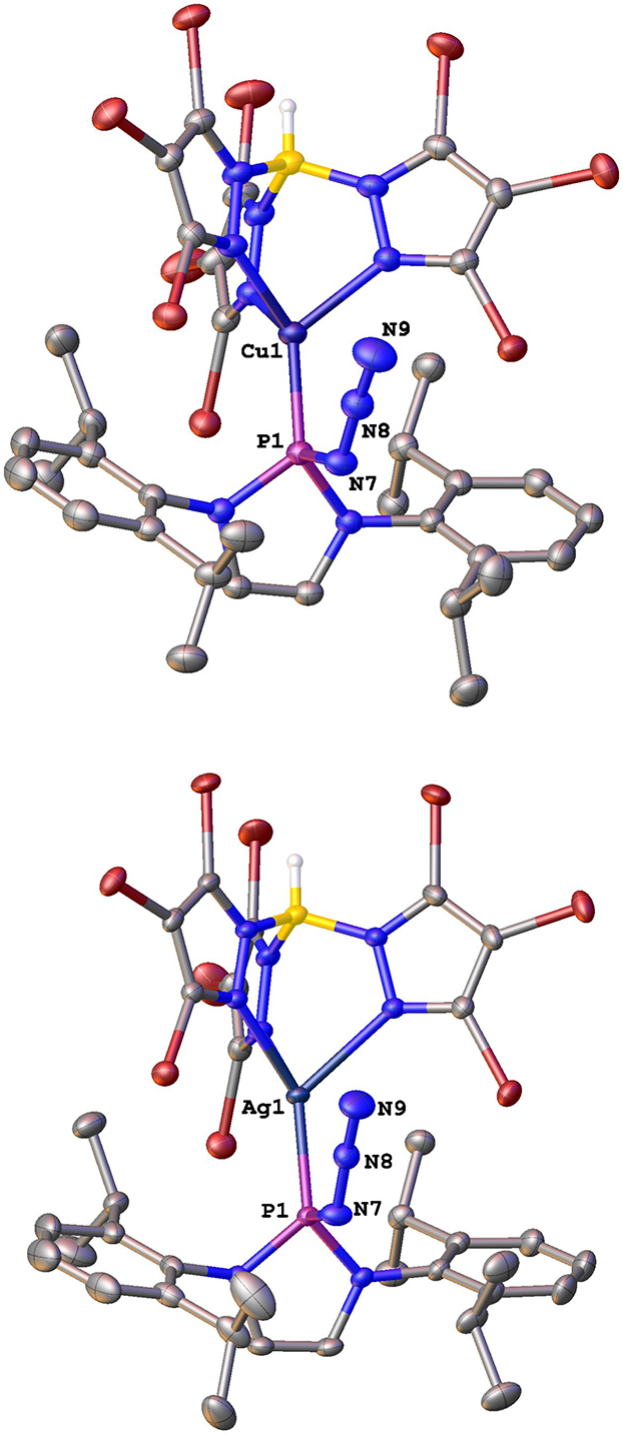
ORTEP diagram of complexes **2** and **3**. Hydrogen atoms are omitted.

Figure 1 contains the solid-state structures of compounds **2** and **3**,^11^ with the phosphorus atom coordinated to the metal center and the azide fragment N3 remaining intact. The main bond lengths are shown in Table 1. For **2**, the Cu–P distance is similar to that reported for copper complexes bearing alkoxydiaminophosphine ligands (2.1601(10) Å).^12^ The same effect is observed for **3**, with the Ag–P bond length very close to that described for the [HB(Pz)_3_]Ag(PPh_3_) complex (2.336(3) Å).^13^ The complexation of **1** to the metal center generates a decrease in the P–N bond length from that of the nonligated phosphinoazide.^14^

**Table 1:** Comparison of the Main Bond Distances (Å) for **1, 2**, and **3**

	bond lengths (Å)		
	azide (**1**)	Cu complex (**2**)	Ag Complex (**3**)
M–P1		2.1569(11)	2.3489(5)
P1–N7	1.834	1.759(4)	1.7595(19)

### Thermal Stability of Complexes **2** and **3**

**Scheme 3:**
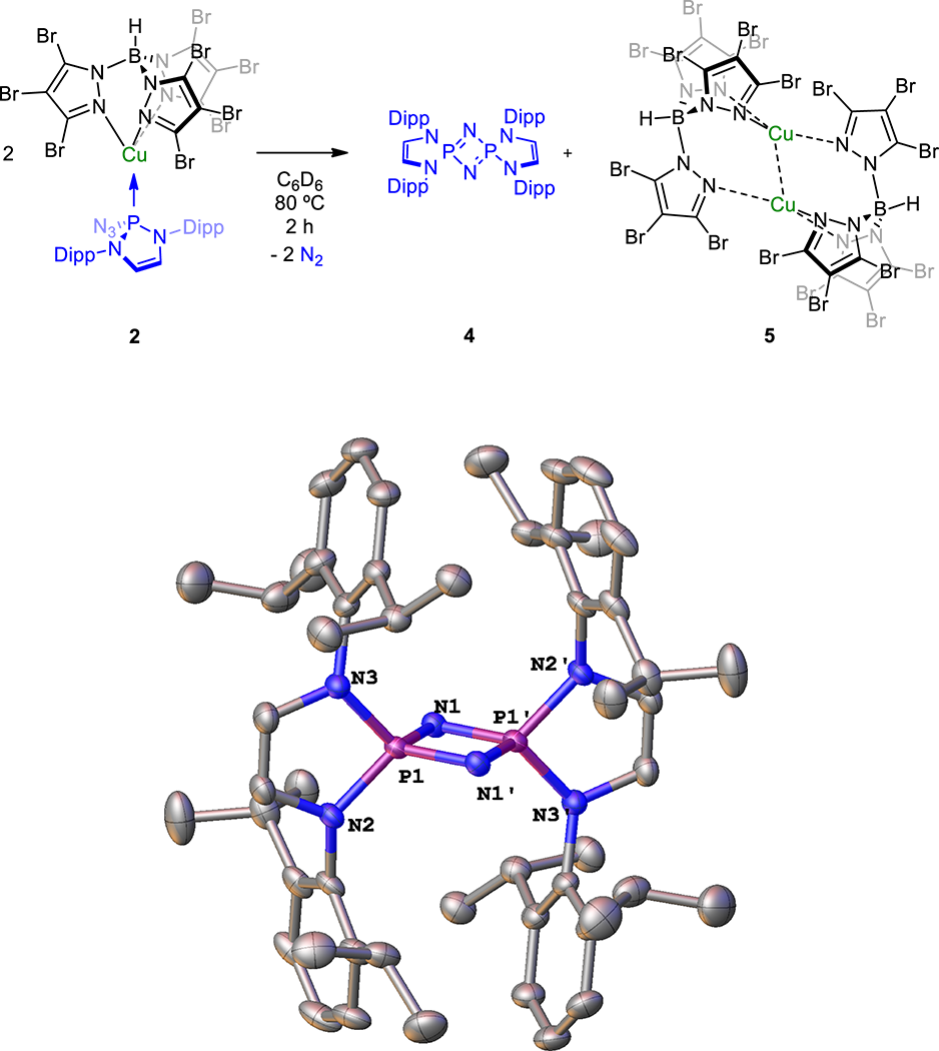
Thermolysis of Complex **2** and X-ray Structure of **4** Hydrogen atoms are omitted for clarity.

Aiming at inducing dinitrogen extrusion and nitrene formation, solutions of both complexes in C_6_D_6_ were heated at 80 °C. In the case of the copper complex, after 2 h of heating, the characteristic signals of **2** were no longer observed in the NMR spectra. Instead, a new set of signals was found, which were assigned to those of λ^5^-cyclodiphosphazene(V) **4** (Scheme 3) previously described from the thermal decomposition of **1**.^15^ This proposal was demonstrated upon crystallization from hexane solutions at −30 °C and determination of its structure by X-ray diffraction studies (Scheme 3).^11^ The P–N distance averages 1.647 Å, being shorter than that for free **1** or those in complexes **2** or **3**. This is in agreement with a certain increase in the P–N bond order, which corresponds to a bond situation between single and double. Furthermore, a weak interaction between both P atoms cannot be discarded since the P–P distance of 2.224 Å is shorter than the sum of the van der Waals radii for both phosphorus atoms (3.6 Å). In addition to **4**, the reaction provides the dinuclear copper complex [Tp^Br3^Cu]_2_ (**5**). This complex presents a structure similar to other binuclear copper and silver complexes such as [Tp*Cu]_2_ or [Tp^Br3^Ag]_2_,^16^ with each copper center bonded to two pyrazolyl rings of one Tp^Br3^ ligand and to a third pyrazolyl ring of the other Tp^Br3^ ligand (see the SI for the X-ray structure of this compound).^11^ A weak copper–copper interaction may exist as inferred from the distance between both ions (2.615 Å), which is smaller than the addition of the van der Waals radii (2.8 Å).

**Figure 2:**
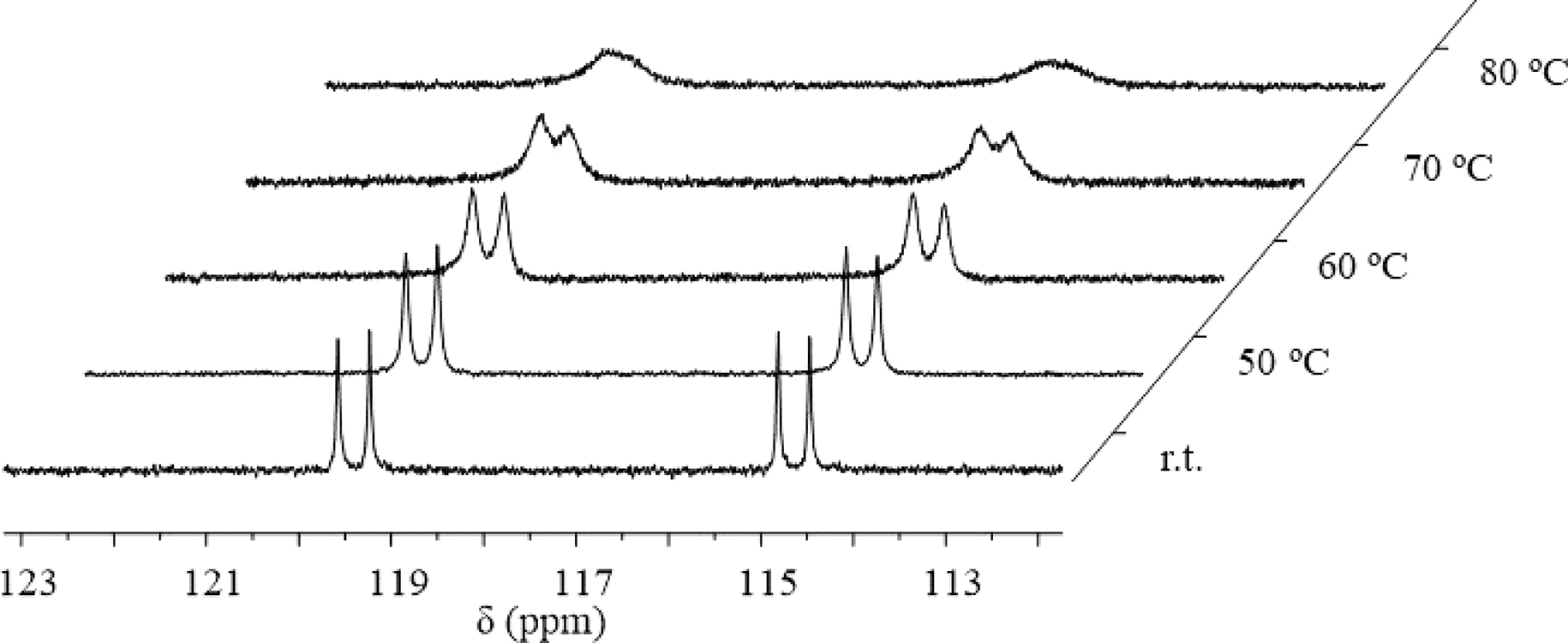
Variable-temperature ^31^P NMR studies for complex **3**.

**Figure 3:**
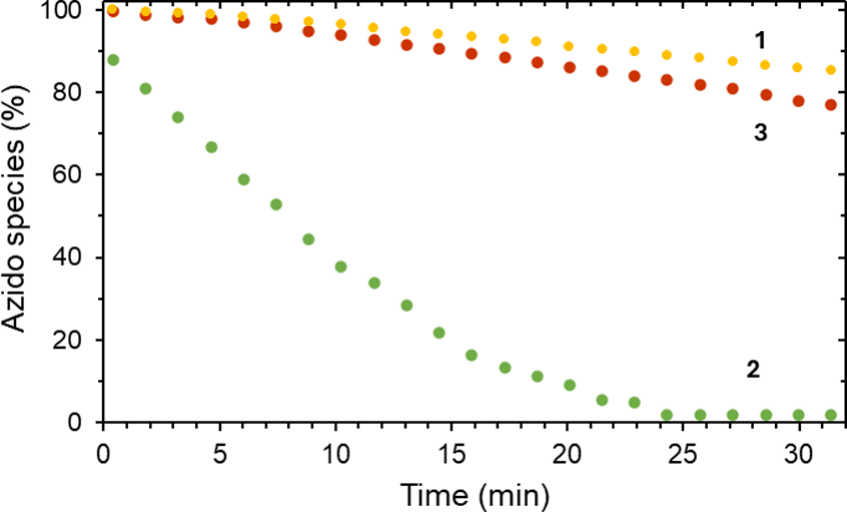
Decomposition of phosphinoazide (**1**), copper- (**2**), and silver complexes (**3**) (toluene-d_8_, 100 °C).

**Scheme 4:**
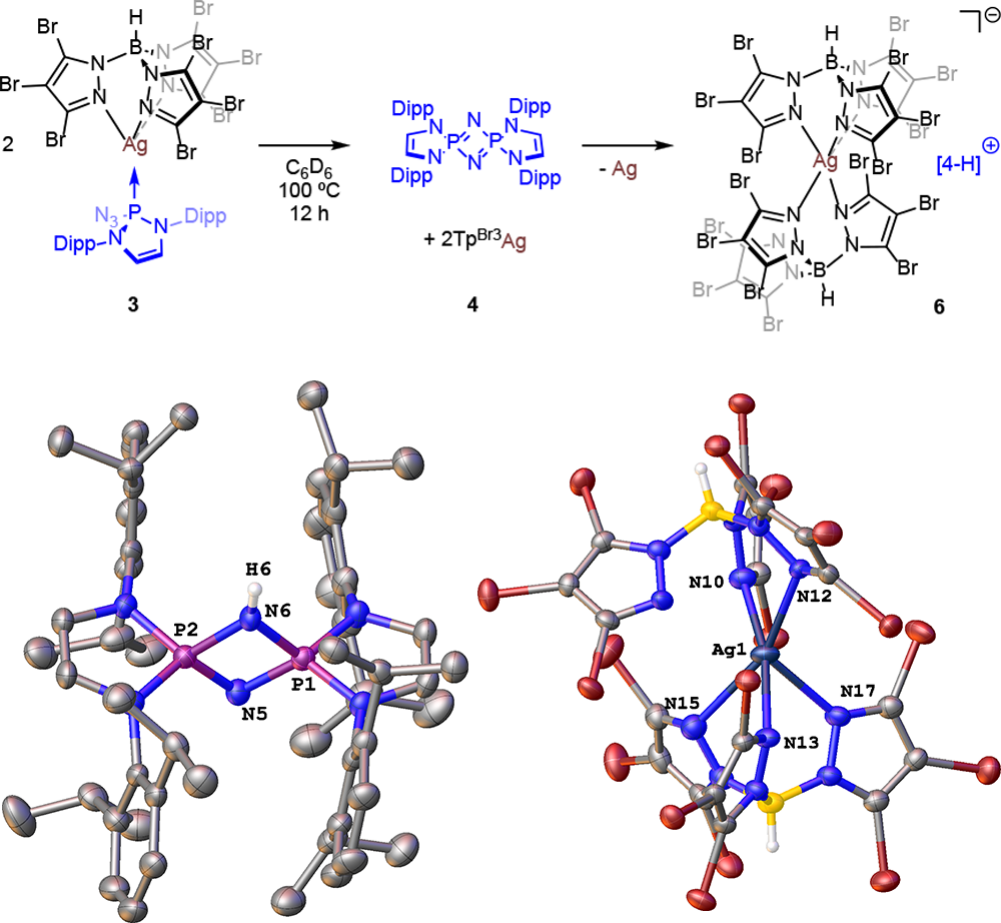
Thermolysis of Complex **3** and the ORTEP Diagram of Complex **6**, Showing the Cation on the Left and the Anion on the Right Hydrogen atoms are omitted for clarity.

At variance with **2**, silver complex **3** showed thermal stability for 2 h at 80 °C, as shown in Figure 2. Although the signals underwent broadening when heating up to that temperature, the initial pattern was recovered upon cooling the sample to room temperature. However, increasing the temperature to 100 °C induced the formation of **4**, which was formed in 30% yield after 12 h at that temperature, when complex **3** was no longer detected by NMR (Scheme 4). The transformation did not stop at **4** since the high temperature used led to the partial decomposition of the Tp^Br3^Ag cores and the formation of an ionic compound of composition [**4**-H]^+^[(κ^3^-Tp^Br3^)[(κ^2^-Tp^Br3^)Ag]® (**6**). This compound was isolated and characterized by X-ray diffraction studies (Figure 3).^11^ Two trispyrazolylborate units are coordinated to a single silver atom in tricoordinate and dicoordinate fashions forming the anion, whereas cyclodiphosphazene **4** is protonated at one of the nitrogen bridging atoms. We are not aware of any previous report for a silver complex bearing two trispyrazolylborate ligands with such coordination modes.

### Kinetic Studies

In view of the different thermal stabilities for **2** and **3**, we decided to carry out kinetic experiments of such a process. Figure 3 shows the ^1^H NMR monitoring of samples of the phosphinoazide (**1**), the copper (**2**), and the silver complexes (**3**) in toluene-d_8_ at 100 °C. From these experiments, it can be concluded that the copper complex presents a remarkably higher decomposition rate than its silver analogue or the free phosphinoazide. In view of the similarity of the behavior for **1** and **3**, it might happen that **3** decoordinates **1**, which undergoes subsequent decomposition.

**Figure 4:**
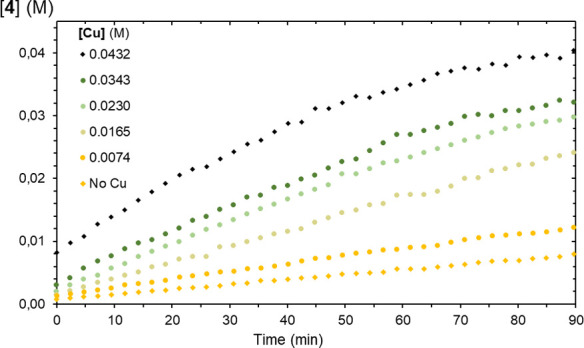
Thermolysis of phosphinoazide (**1**), in the presence of variable concentrations of Tp^Br3^Cu(NCMe) (toluene-d_8_, 80 °C).

**Figure 5:**
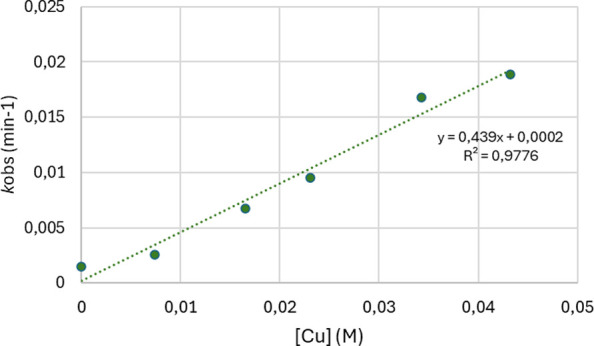
Correlation of *k*_obs_ with [Tp^Br3^Cu(NCMe)].

Next, we explored the effect of the added Tp^Br3^Cu(NCMe) on solutions of phosphinoazide (**1**). As depicted in Figure 4, a gradual increment in the rate of formation of **4** was found when increasing the concentration of the copper complex, which was employed in substoichiometric amounts in all cases. Figure 5 plots the concentrations of Cu employed and the resulting observed rate constants (see the SI for details), leading to a quasilinear correlation with *R*^2^ = 0.9777 (Figure 5).

To the best of our knowledge, the formation of λ^5^-cyclodiphosphazene(V) (**4**) has been reported exclusively by means of photochemical or thermal decomposition of azides, the latter requiring heating of at least 110 °C for several hours.^15^ Since *k*_obs_ in the absence of copper is neither zero nor negligible, it can be proposed that two different routes (copper-catalyzed and thermal azide decomposition) lead to the formation of the cyclodiphosphazene in these conditions (see below).

### Mechanistic Proposal

**Scheme 5:**
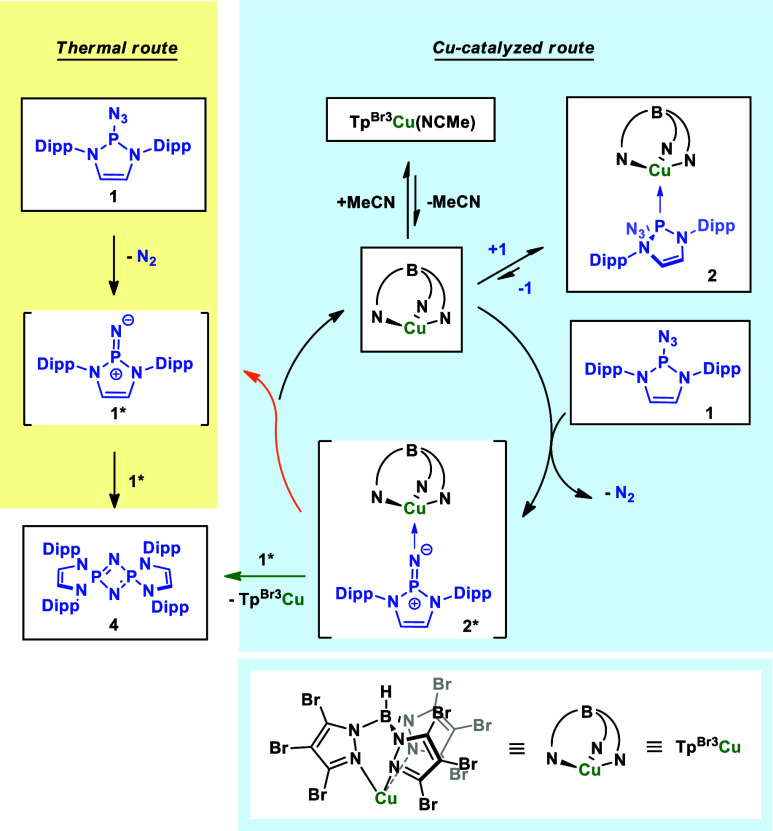
Proposed Mechanism for the Formation of **4** by Tp^Br3^Cu(NCMe)

Since the generation of compound **4** in a catalytic manner is yet unknown, a plausible mechanistic explanation is herein provided (Scheme 5). Given that **1** provides **4** upon thermal decomposition, a metal-free route must be considered (albeit it is not the most productive one), as previously proposed.^5,17^ This thermal route would start with extrusion of N_2_ from **1**, generating the free nitrene 1*, which undergoes a [2 + 2] cycloaddition reaction with a second nitrene unit via a zwitterionic intermediate.

In the metal-catalyzed route, the complex Tp^Br3^Cu(NCMe) reacts with **1** affording **2**, via a coordinatively and electronically unsaturated fragment Tp^Br3^Cu. This is an equilibrium lying at **2**, since a solution of equimolar concentrations of phosphinoazide (**1**) and the Tp^Br3^Cu(NCMe) complex in toluene-d_8_ shows that only 1% of the azide was free in solution. However, we propose that **2** is an off-cycle species since it does not present the correct coordination for an effective N_2_ extrusion and it would act as a Tp^Br3^Cu reservoir, as observed for other Cu-based systems for carbene transfer.^18^ Coordination of **1** to the Tp^Br3^Cu fragment through the nitrogen atom facilitates nitrogen extrusion and provides metallonitrene 2*, which constitutes the rate-determining step. We have no data to propose whether the dimerization process takes place between 2* and 1* (green route) or via a nitrene-decoordination step (orange route). However, due to the high steric hindrance in 2*, we think that the latter could be more favorable. Additional attempts at the isolation of compound 2* were not fruitful.

This proposal also explains the lower decomposition rate when using complex **3** since the generation of Tp^Br3^Ag is more difficult due to the higher affinity between P(III) (a soft base) and Ag(I) (a soft acid). This is reflected in the NMR studies at high temperatures (Figure 2), in which free phosphinoazide is never observed. It is also important to note that examples of silver complexes able to decompose azides are scarce.^19^

## Conclusions

Two complexes containing the Tp^Br3^M (M = Cu, Ag) core and a phosphinoazide ligand have been synthesized. Their thermal decomposition has been studied, in which λ^5^-cyclodiphosphazene(V) (**4**) is formed. We have found that with catalytic amounts of the copper complex, the reaction takes place at a higher rate than the sole thermal decomposition of **1**, in the first example of this process taking place in a catalytic manner employing phospinoazides.

## Experimental Section

### General Considerations

To ensure the stability of all of the compounds used, all of the preparations and manipulations were carried out under strictly anhydrous conditions and in the absence of oxygen using conventional Schlenk techniques or using an inert chamber (MBRAUN UNILAB). The solvents employed were dried before use by distillation with the appropriate desiccant, deoxygenated by passing a current of N_2_ for 10 min, and stored in a Teflon-tapped ampule. The reagents used were purchased from Aldrich or Alfa Aesar and used without any prior purification. The complexes Tp^Br3^Cu(NCMe) and [Tp^Br3^Ag]_2_ were synthesized by procedures described in the literature.^20^ Azide **1** was synthesized from 2-bromo-1,3-bis(2,6-diisopropylphenyl)-2,3-dihydro-1*H*-1,3,2-diazaphosphole.^21^ NMR spectra were recorded on Agilent 400MR and 500DD2. The chemical shifts of the ^1^H and ^13^C spectra are referenced for tetramethylsilane, using the signal of the deuterated solvent as an internal reference. The elemental analyses were carried out in a PerkinElmer Series II CHNS/O Analyzer 2400 elemental analyzer. X-ray diffraction experiments were carried out at the CIQSO-UHU.

#### Synthesis of Azido-1,3-bis(2,6-diisopropylphenyl)-2,3-dihydro-1*H*-1,3,2-diazaphosphole (**1**)

2-Bromo-1,3-bis(2,6-diisopropylphenyl)-2,3-dihydro-1*H*-1,3,2-diazaphosphole (4.8 g, 10 mmol) was introduced into a Schlenk flask equipped with a magnetic stirrer and dissolved in 30 mL of THF. Then, NaN_3_ (0.99 g, 15 mmol) was subsequently added, followed by LiCl (0.1 g, 2.35 mmol), and the mixture was stirred for 8 h at room temperature. Volatiles were removed under reduced pressure to give a beige solid, which was extracted with hexane and filtered via a cannula (4 × 20 mL). The resulting solution was stored at −33 °C for 5 days. The crystals obtained were filtered and dried under vacuum, obtaining azido-1,3-bis(2,6-diisopropylphenyl)-2,3-dihydro-1*H*-1,3,2-diazaphosphole) **1** (2.3 g, 51% yield). ^1^H NMR (400 MHz, C_6_D_6_): δ 7.24–7.16 (m, 2H, C-*H*_Ar_), 7.14–7.06 (m, 4H, C-*H*_Ar_), 5.89 (d, 2H, ^3^*J*_H–P_ = 1.5 Hz, C-*H*_cycle_), 3.92–3.79 (m, 2H, C-*H*_Dipp_), 3.36–3.25 (m, 2H, C-*H*_Dipp_), 1.30 (d, 12H, ^3^*J* = 6.9 Hz, C-*H*_3Dipp_), 1.20–1.10 (m, 12H, C-*H*_3Dipp_). ^13^C{^1^H} NMR (100 MHz, C_6_D_6_): δ 149.4 (*C*_Ar_), 147.7 (*C*_Ar_), 135.4 (d, ^2^*J*_C–P_ = 12.8 Hz, *C*_Ar_), 129.0 (d, ^2^*J*_C–P_ = 1.8 Hz, *C*-H_Ar_), 125.1 (*C*-H_Ar_), 124.2 (*C*-H_Ar_), 119.6 (d, ^3^*J*_C–P_ = 7.5 Hz, *C*-H_cycle_), 29.1 (*C*-H_3Dipp_), 25.2 (*C*-H_3Dipp_), 24.9 (*C*-H_Dipp_), 24.2 (*C*-H_Dipp_). ^31^P{^1^H} NMR (162 MHz, C_6_D_6_): δ 120.7.

#### Synthesis of Complex **2**

In a Schlenk tube equipped with a magnetic stirrer, the Tp^Br3^Cu(NCMe) complex (103 mg, 0.1 mmol) was dissolved in 5 mL of DCM, and a solution of azide **1** (68 mg, 0.15 mmol) in 5 mL of DCM was added via a cannula. The solution was stirred for 30 min, followed by the addition of 8 mL of hexane. The solution was concentrated by dynamic vacuum until cloudiness. After 2 h, crystals were separated and dried under a vacuum, obtaining complex **2**, which crystallizes from hexane-DCM solutions (116 mg, 80% yield). ^1^H NMR (500 MHz, C_6_D_6_): δ 7.15–7.10 (m, 2H, C-*H*_Ar_), 7.07–7.04 (m, 2H, C-*H*_Ar_), 7.01–6.96 (m, 2H, C-*H*_Ar_), 5.82 (d, 2H, ^3^*J*_H–P_ = 12.2 Hz, C-*H*_cycle_), 5.59 (br s, 1H, B-*H*), 4.11 (hept, 2H, ^3^*J* = 6.8 Hz, C-*H*_Dipp_), 3.66 (hept, 2H, ^3^*J* = 6.8 Hz, C-*H*_Dipp_), 1.37 (d, 6H, ^3^*J* = 6.8 Hz, C-*H*_3Dipp_), 1.17 (d, 6H, ^3^*J* = 6.8 Hz, C-*H*_3Dipp_), 1.09 (d, 6H, ^3^*J* = 6.8 Hz, C-*H*_3Dipp_), 0.69 (d, 6H, ^3^*J* = 6.8 Hz, C-*H*_3Dipp_). ^13^C{^1^H} NMR (125 MHz, C_6_D_6_): δ 150.3 (d, ^3^*J*_C–P_ = 3.8 Hz, *C*_Ar_), 149.2 (d, ^2^*J*_C–P_ = 4.6 Hz, *C*_Ar_), 134.5 (*C*_Ar_), 131.2 (*C*_Pz_), 131.0 (*C*_Pz_), 130.0 (*C*-H_Ar_), 125.3 (*C*-H_Ar_), 124.1 (*C*_Pz_), 123.6 (*C*_*Pz*_), 119.7 (d, ^3^*J*_C–P_ = 2.8 Hz, *C*-H_cycle_), 101.5 (*C*_Pz_), 101.3 (*C*_Pz_), 29.6 (*C*-H_Dipp_), 29.1 (*C*-H_Dipp_), 27.5 (*C*-H_3Dipp_), 26.2 (*C*-H_3Dipp_), 24.5 (*C*-H_3Dipp_), 21.5 (*C*-H_3Dipp_). ^31^P{^1^H} NMR (162 MHz, C_6_D_6_): δ 108.0. ^11^B{^1^H} NMR (128 MHz, C_6_D_6_): δ −5.7. IR (nujol): 2120 cm^–1^ (N3). Elemental analysis calcd for C_35_H_37_BBr_9_CuN_11_P·0.65C_6_H_12_: C, 31.30; H, 3.16; N, 10.32. Found: C, 31.85; H, 3.12; N, 10.14.

#### Synthesis of Complex **3**

In a Schlenk tube equipped with a magnetic stirrer and covered with aluminum foil, the [Tp^Br3^Ag]_2_ complex (103 mg, 0.05 mmol) was dissolved in 5 mL of DCM. Then, a solution of azide **1** (68 mg, 0.15 mmol) in 5 mL of DCM was added via a cannula. The reaction was stirred for 30 min at room temperature. Then, the solution was filtered via a cannula and 8 mL of hexane was added. Upon concentration until cloudiness, crystals appeared after 2 h, which were separated and dried under a vacuum, affording complex **3** (110 mg, 75% yield). ^1^H NMR (500 MHz, C_6_D_6_): δ 7.15–7.10 (m, 2H, C-*H*_Ar_), 7.07–7.02 (m, 4H, C-*H*_Ar_), 5.82 (d, 2H, ^3^*J*_H–P_ = 10.5 Hz, C-*H*_cycle_), 5.60 (br s, 1H, B-*H*), 3.96 (hept, 2H, ^3^*J* = 6.8 Hz, C-*H*_Dipp_), 3.53 (hept, 2H, ^3^*J* = 6.8 Hz, C-*H*_Dipp_), 1.30 (d, 6H, ^3^*J* = 6.8 Hz, C-*H*_*3*Dipp_), 1.15–1.10 (m, 18H, C-*H*_*3*Dipp_). ^13^C{^1^H} NMR (125 MHz, C_6_D_6_): δ 149.9 (d, ^*3*^*J*_*C–P*_ = 3.8 Hz, *C*_Ar_), 148.3 (d, ^2^*J*_C–P_ = 4.4 Hz, *C*_Ar_), 133.1 (*C*_Ar_),130.7 (*C*_Pz_), 130.1 (*C*-H_Ar_), 125.5 (*C*-H_Ar_), 125.4 (*C*-H_Ar_), 124.2 (*C*_Pz_), 119.8 (t, ^3^*J*_C–P_ = 4.0 Hz, ^4^*J*_C–Ag_ = 4.0 Hz, *C*-H_cycle_), 100.7 (*C*_Pz_), 29.9 (*C*-H_Dipp_), 29.3 (*C*-H_Dipp_), 26.1 (*C*-H_3Dipp_), 25.9 (*C*-H_3Dipp_), 24.6 (*C*-H_3Dipp_), 22.4 (*C*-H_3Dipp_). ^31^P{^1^H} NMR (202 MHz, C_6_D_6_): δ 116.9 (d, ^1^*J*_Ag(107)–P_ = 1030.2 Hz), 116.8 (d, ^1^*J*_Ag(109)–P_ = 888.8 Hz). ^11^B{^1^H} NMR (128 MHz, CD_2_Cl_2_): δ −5.2. IR (nujol): 2120 cm^–1^ (N3). Elemental analysis calcd for C_35_H_37_BBr_9_AgN_11_P: C, 28.39; H, 2.52; N, 10.41. Found: C, 28.85; H, 2.62; N, 9.97.

#### Synthesis of Cyclodiphosphazene **4**

Azide **1** (250 mg, 0.56 mmol) was placed into a Teflon-tapped ampule equipped with a magnetic stirrer, and 10 mL of dry and deoxygenated toluene was added. The ampule was heated in an oil bath at 110 °C for 2 h. After this time, volatiles were removed under reduced pressure and 10 mL of hexane was added. The solution was transferred to a Schlenk tube via a cannula and kept at −33 °C. After 24 h, colorless crystals appeared, which were filtered and dried under vacuum, leading to cyclodiphosphazene **4** (150 mg, 60% yield). ^1^H NMR (400 MHz, C_6_D_6_): δ 7.12 (t, *J* = 7.7 Hz, 4H, C-*H*_Ar_), 6.97 (d, *J* = 7.7 Hz, 8H, C-*H*_Ar_), 5.44–5.39 (m, 4H, C-*H*_cycle_), 3.47 (hept, *J* = 6.8 Hz, 8H, C-*H*_Dipp_), 1.22 (d, *J* = 6.8 Hz, 24H, C-*H*_3Dipp_), 1.17 (d, *J* = 6.8 Hz, 24H, C-*H*_3Dipp_). ^13^C{^1^H} NMR (100 MHz, C_6_D_6_): δ 148.7 (*C*_Ar_), 135.8 (*C*_Ar_), 128.6 (*C*-H_*Ar*_), 124.5 (*C*-H_Ar_), 118.7 (t, *J* = 7.6 Hz, *C*-H_cycle_), 28.7 (*C*-H_Dipp_), 25.7 (*C*-H_3Dipp_), 23.6 (*C*-H_3Dipp_). ^31^P{^1^H} NMR (162 MHz, C_6_D_6_): δ 34.8. Elemental analysis calcd for C_52_H_72_N_6_P_2_: C, 74.08; H, 8.61; N, 9.97. Found: C, 74.44; H, 9.48; N, 9.89.

#### Thermal Decomposition of Complex **2**

In a Schlenk tube equipped with a magnetic stirrer, complex **2** (100 mg, 0.069 mmol) was added, followed by 3 mL of dry and deoxygenated benzene. The tube was heated in an oil bath at 80 °C for 2 h. Upon cooling, colorless crystals of complex [Tp^Br3^Cu]_2_ (**5**) appeared, which were separated by filtration under N_2_ and washed twice with 5 mL of hexane (50 mg, 73% yield). The filtrate was evaporated to dryness and dissolved in deuterobenzene; 1,3,5-trimethoxybenzene was added as an internal standard, and the solution was analyzed by ^1^H NMR, demonstrating the formation of **4** in 85% yield.

For the NMR-scale Thermolysis reaction of **2**, complex **2** was introduced into a J-Young NMR tube (30 mg, 0.02 mmol) and dissolved in 0.5 mL of benzene-d_6_ inside a dry box. The sample was heated in an oil bath at 80 °C for 2 h and subsequently analyzed by ^1^H NMR.

#### Characterization of Complex **5**

Due to its low solubility in most noncoordinating deuterated solvents, characterization of complex **5** using NMR techniques has not been possible. X-ray diffraction studies led to its molecular structure.^11^ Elemental analysis calcd for C_18_H_2_B_2_Br_18_Cu_2_N_12_·0.6C_6_H_6_: C, 12.84; H, 0.28; N, 8.32. Found: C, 13.02; H, 0.31; N, 8.12. IR (nujol): 2572 cm^–1^ (B–H).

#### Thermal Stability of Complex **3**

In a J-Young NMR tube, complex **3** (30 mg, 0.02 mmol) was added followed along with 0.5 mL of benzene-d_6_. The sample was heated in an oil bath at 100 °C for 12 h. Monitoring by ^1^H NMR showed the formation of **4** in 30% yield along with some insoluble material. The solution provided a crystalline material upon standing at room temperature for complex **6**.

#### Kinetic Experiments for the Thermolysis of **1**,**2**, and **3**

In a J-Young NMR tube, 0.005 mmol of compounds **1, 2,** or **3** was dissolved in 0.5 mL of toluene-d_8_. The sample was monitored inside the NMR instrument at 100 °C for 35 min.

#### Kinetic Experiments for the Thermolysis of Phosphinoazide **1** in the Presence of the Complex Tp^Br3^Cu(NCMe)

In a J-Young NMR tube, compound **1 (**21.5 mg, 0.048 mmol), the corresponding amount of the Tp^Br3^Cu(NCMe) complex, and 1,3,5-trimethoxybenzene (9.5 mg, 0.0565 mmol) were dissolved in 0.5 mL of toluene-d_8_. The samples were monitored by NMR at 80 °C for 2 h. The values of *k*_obs_ were obtained from an e^*–xt*^ fitting of [**1**] vs time during the first 20 min of the reaction.

## Supporting Information Available

Multinuclear (^1^H, ^13^C, ^31^P, and ^11^B) NMR spectra for new compounds, and kinetic data for decomposition of **1** (PDF)
