# Supplementary material for: Copper and Silver Trispyrazolylborate-Phosphinoazide Complexes: Synthesis, Characterization, and Nitrene Generation
Source: Inorg Chem. 2025 Jan 2;64(1):151–7. doi: 10.1021/acs.inorgchem.4c04397 (PMC11734125; doi:10.1021/acs.inorgchem.4c04397)

SUPPORTING INFORMATION FOR

Copper and Silver Trispyrazolylborate-Phosphinoazide Complexes: Synthesis, Characterization and Nitrene Generation

Manuel R. Rodríguez, Francisco Molina, M. Mar Díaz-Requejo* and Pedro J. Pérez*

Laboratorio de Catálisis Homogénea, Unidad Asociada al CSIC, CIQSO-Centro de Investigación en Química Sostenible and Departamento de Química, Universidad de Huelva, 21007 Huelva, Spain.

1-Kinetic experiment for the decomposition of phosphinoazide **1** in the presence of the complex $Tp^{Br3}Cu(NCMe)$. **S2**

2- NMR spectra **S3**

Table S1. Thermolysis of phosphinoazide (**1**), in the presence of variable concentrations of $Tp^{Br3}Cu(NCMe)$ (toluene-d_6, 80ºC). Initial concentration of phosphinoazide **1** = 0.0956 M

$Tp^{Br3}Cu(NCMe)$ mol (%)	$Tp^{Br3}Cu(NCMe)$ [M]	k_{obs} (min^{-1})	Yield (%)
0.0	0.0000	0.001491	18
7.7	0.0074	0.002587	32
17.2	0.0165	0.006779	54
24.0	0.0231	0.009514	69
35.7	0.0343	0.016750	69
45.0	0.0432	0.018904	79

2- NMR spectra.

^{1}H NMR spectrum of **1** (400 MHz, C_6D_6)

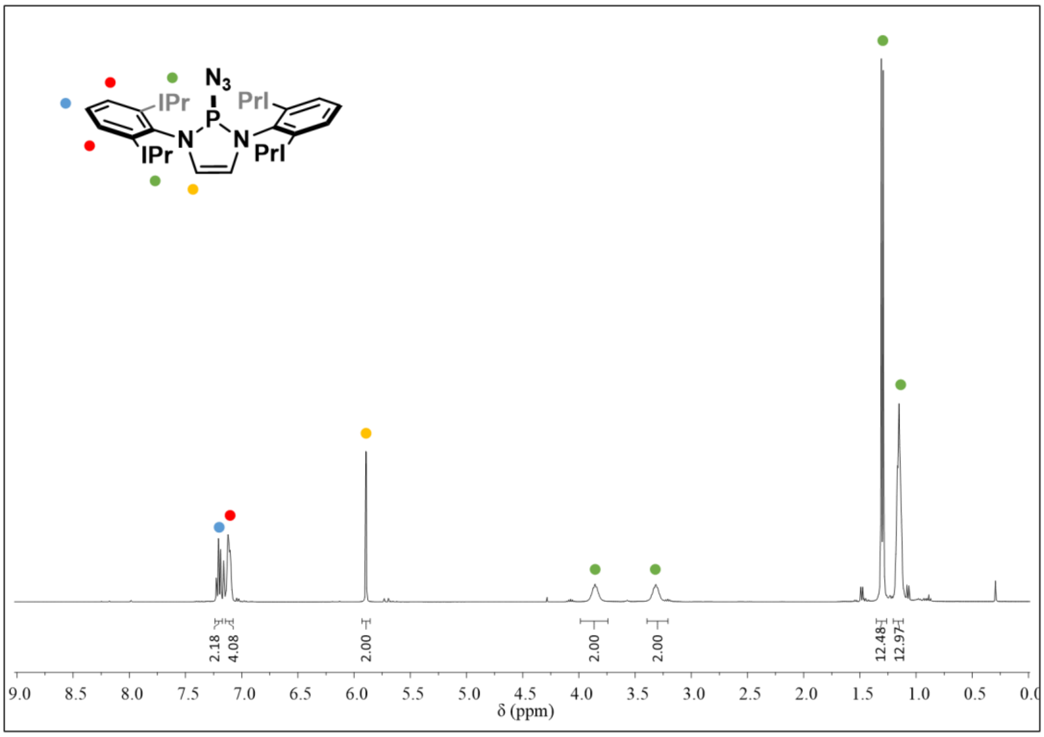

$^{13}C\{^{1}H\}$ NMR spectrum of **1** (100 MHz, C_6D_6)

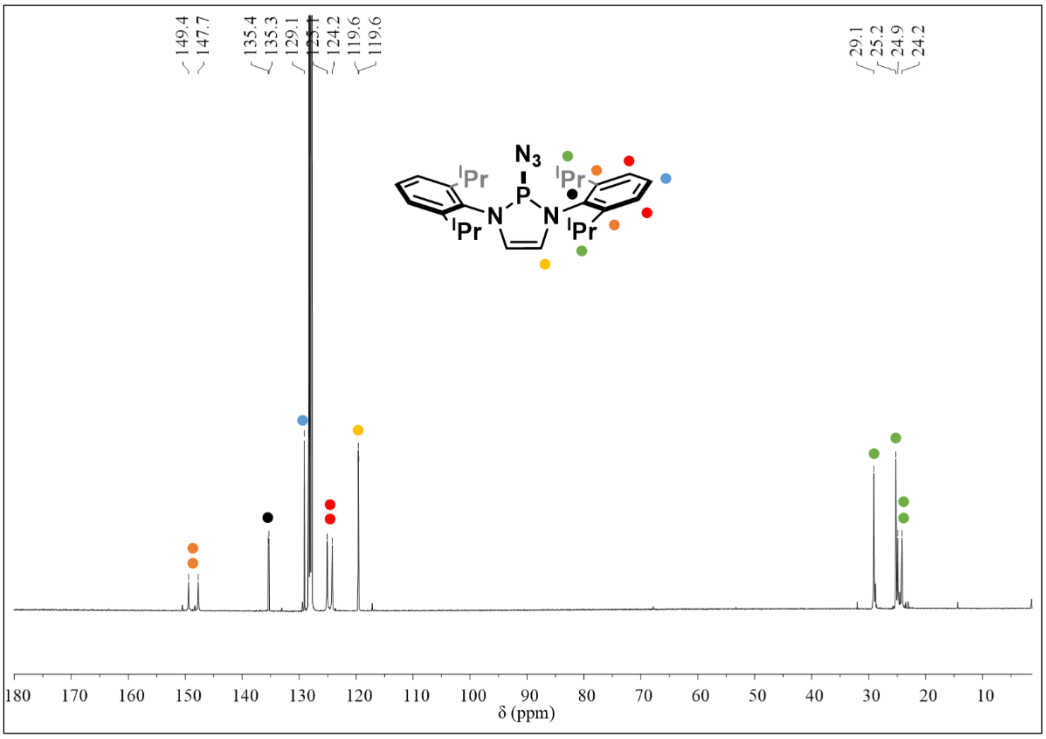

^{1}H NMR spectrum of **2** (500 MHz, C_6D_6)

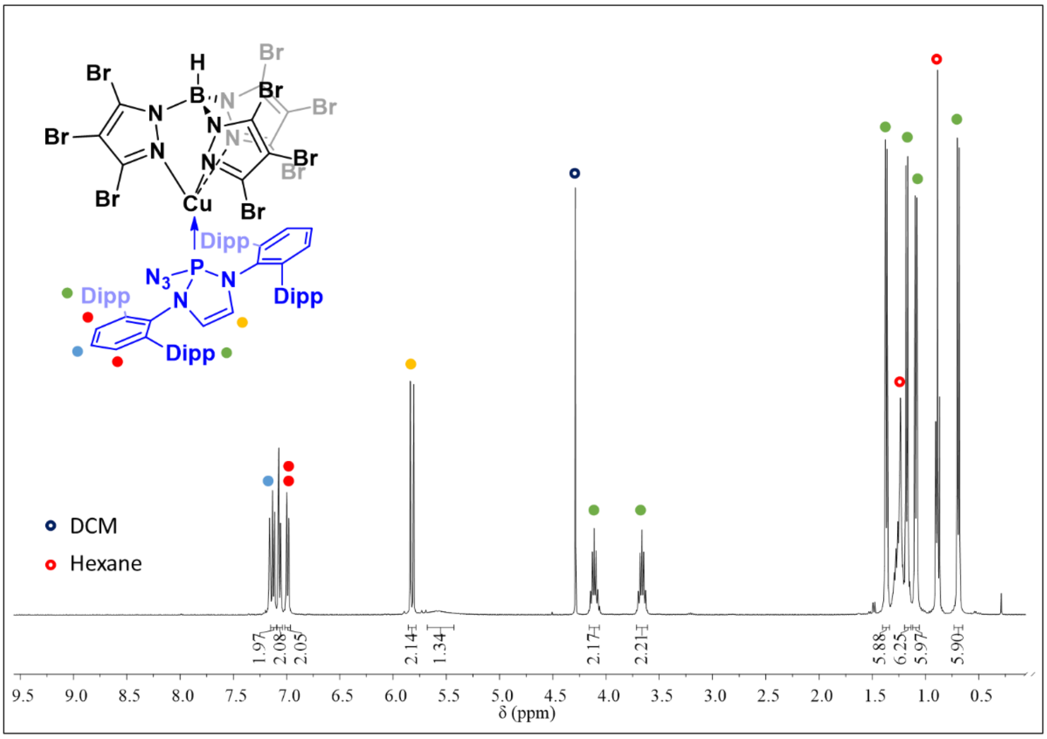

$^{13}C\{^{1}H\}$ NMR spectrum of **2** (125 MHz, C_6D_6)

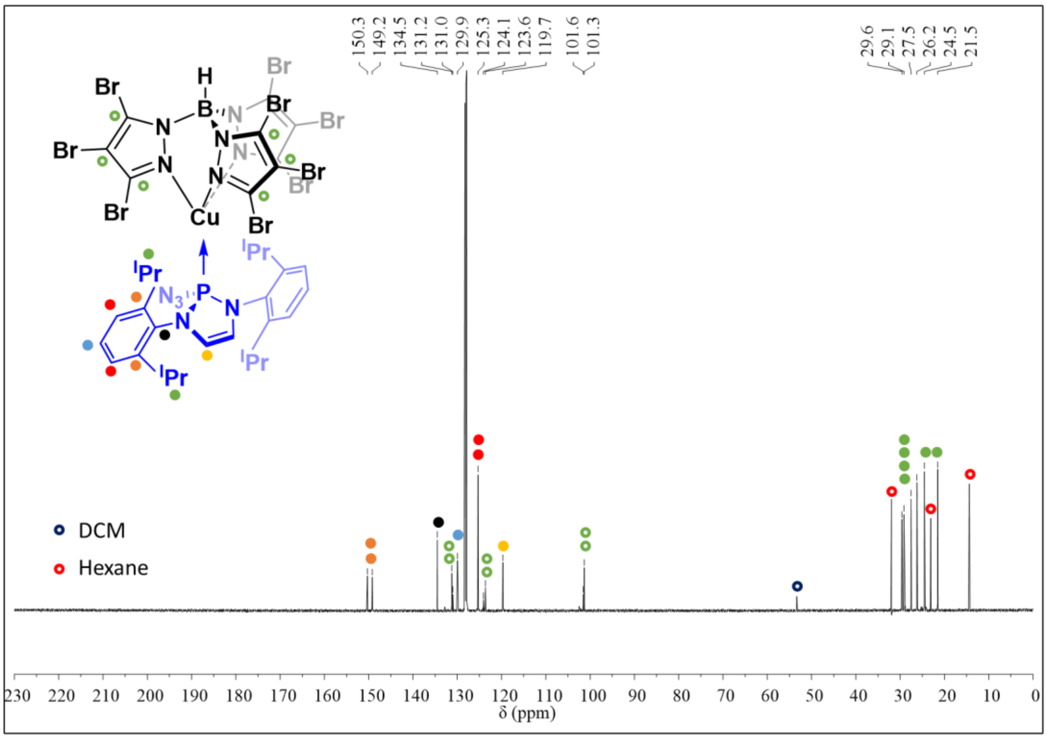

$^{31}P\{^{1}H\}$ NMR spectrum of **2** (162 MHz, C_6D_6)

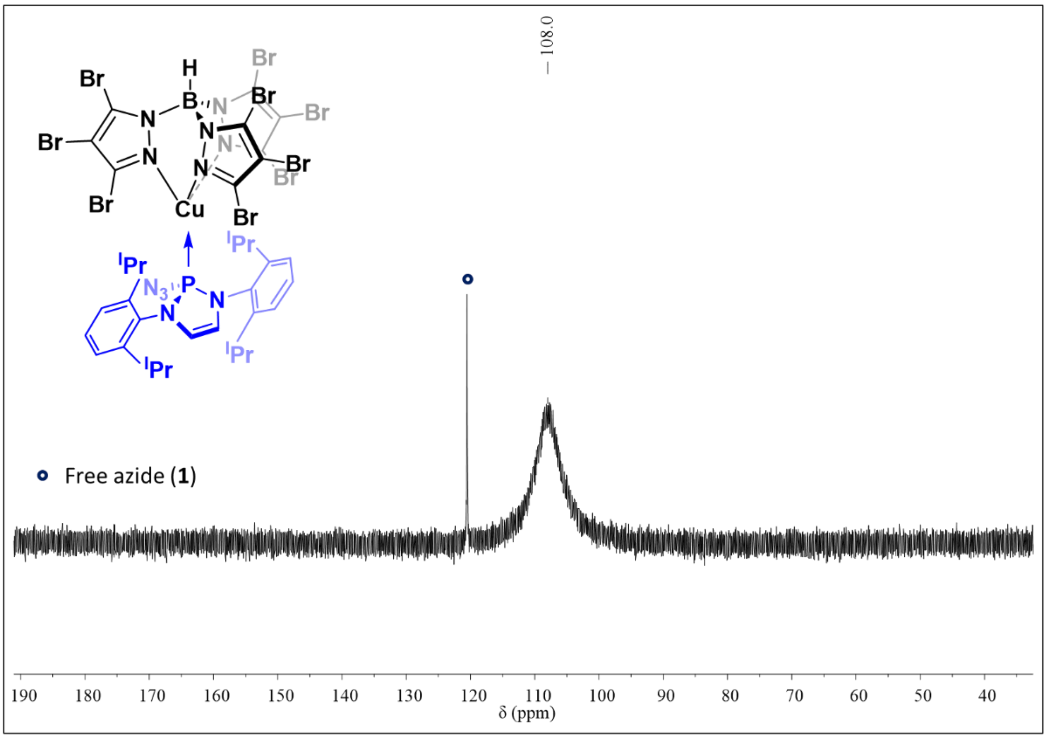

$^{11}B\{^{1}H\}$ NMR spectrum of **2** (128 MHz, C_6D_6)

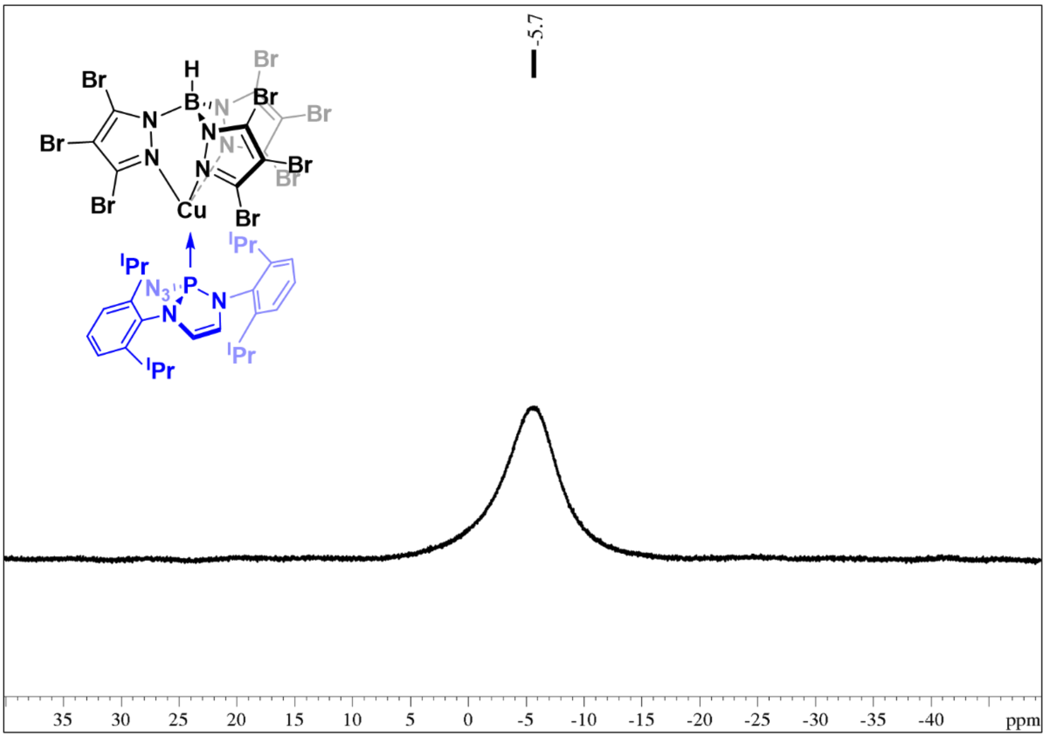

^{1}H NMR spectrum of **3** (500 MHz, C_6D_6)

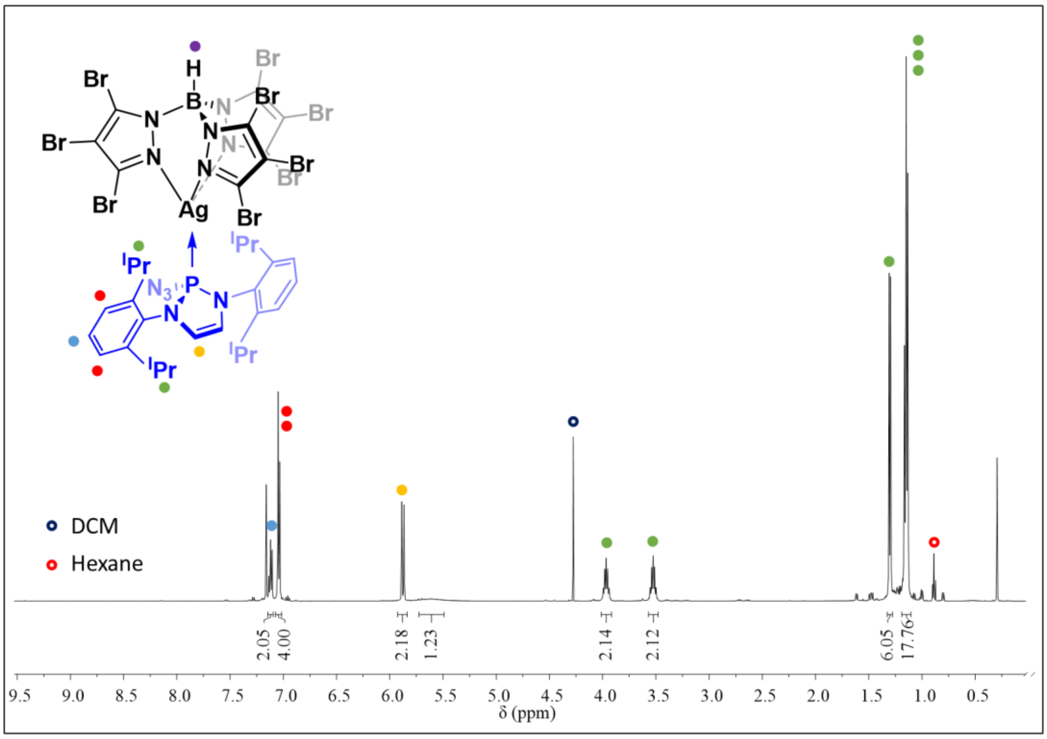

$^{13}C\{^{1}H\}$ NMR spectrum of **3** (125 MHz, C_6D_6)

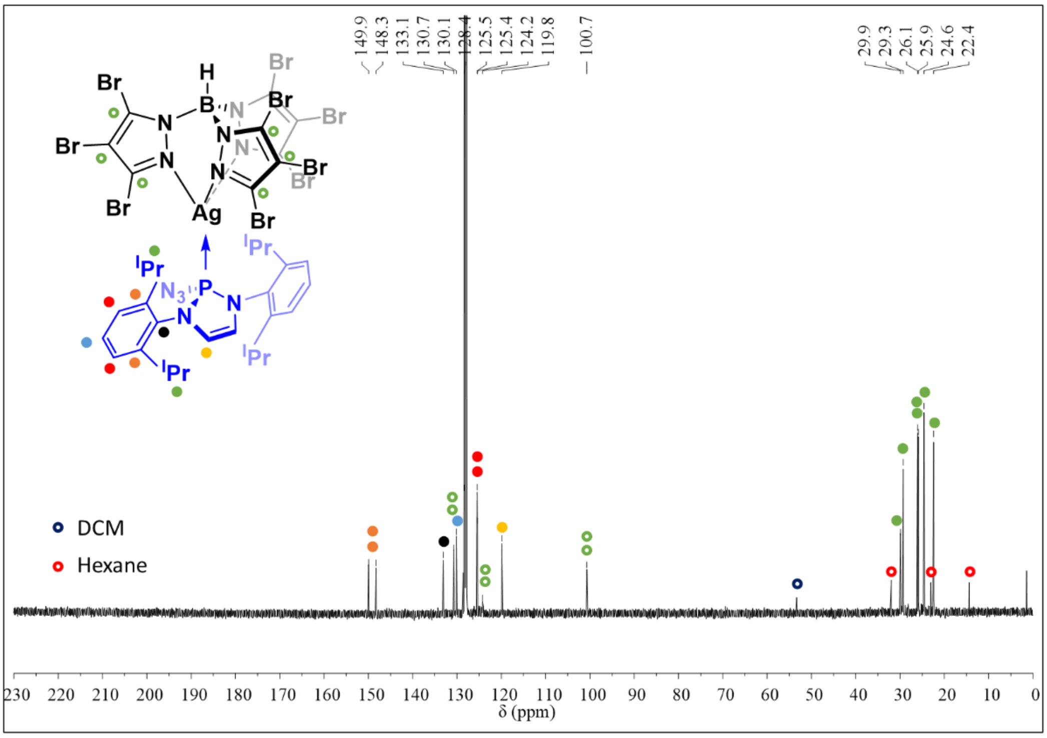

$^{31}P\{^1H\}$ NMR spectrum of **3** (202 MHz, C_6D_6)

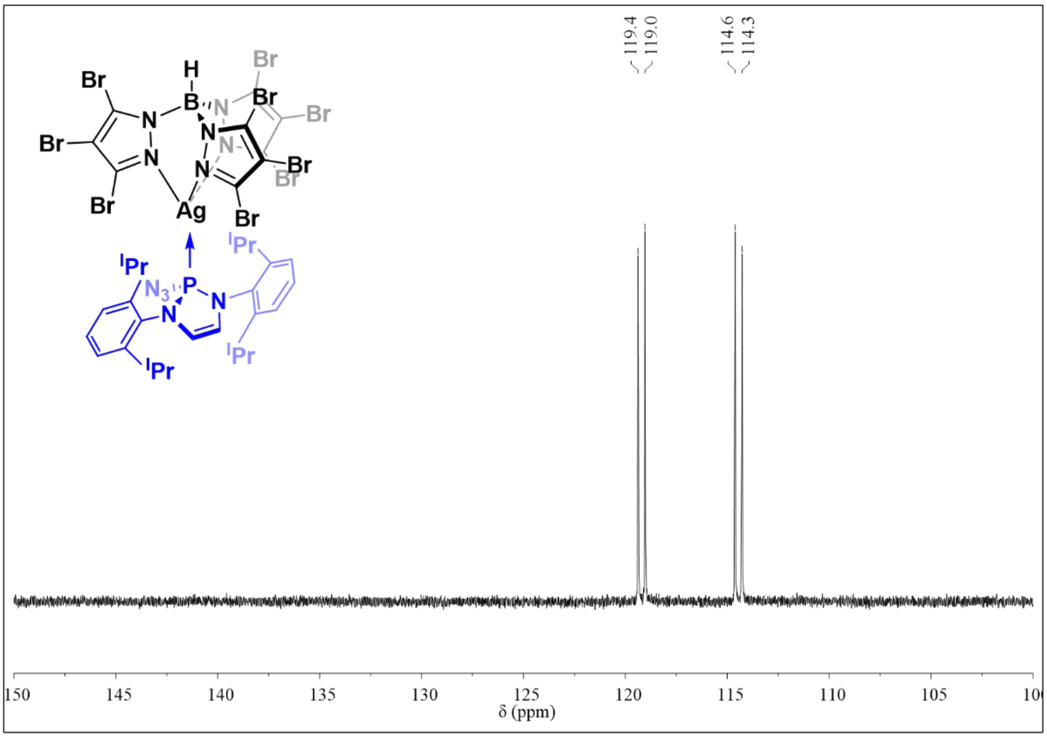

$^{11}B\{^1H\}$ NMR spectrum of **2** (128 MHz, C_6D_6)

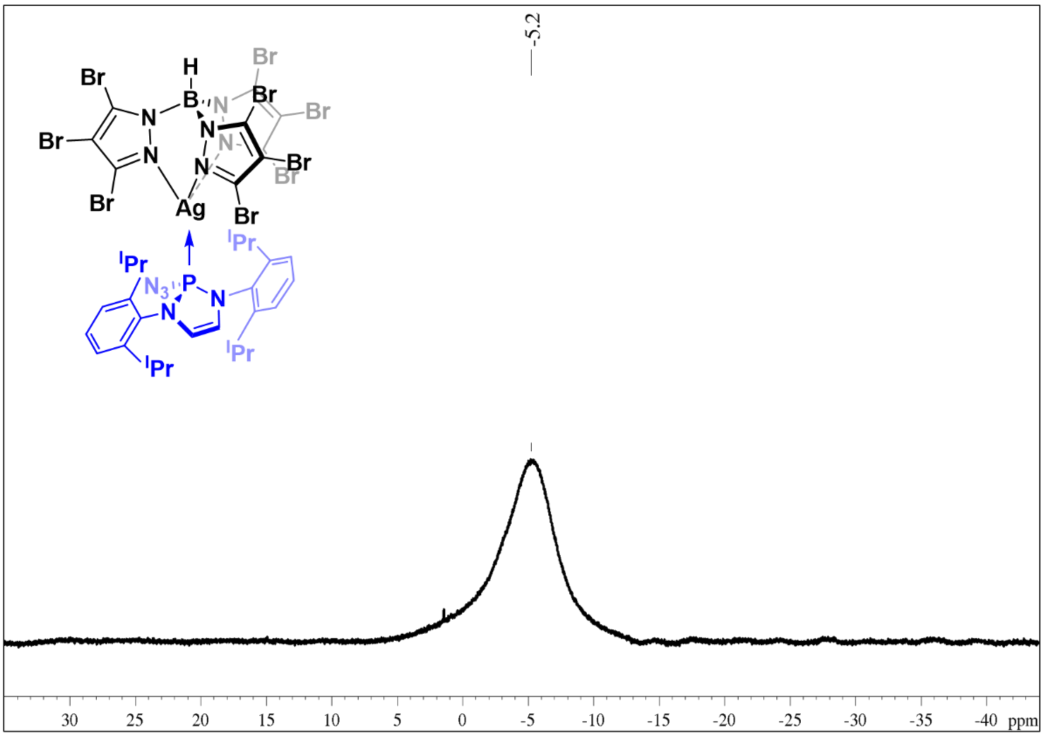

^{1}H NMR spectrum of **4** (400 MHz, C_6D_6)

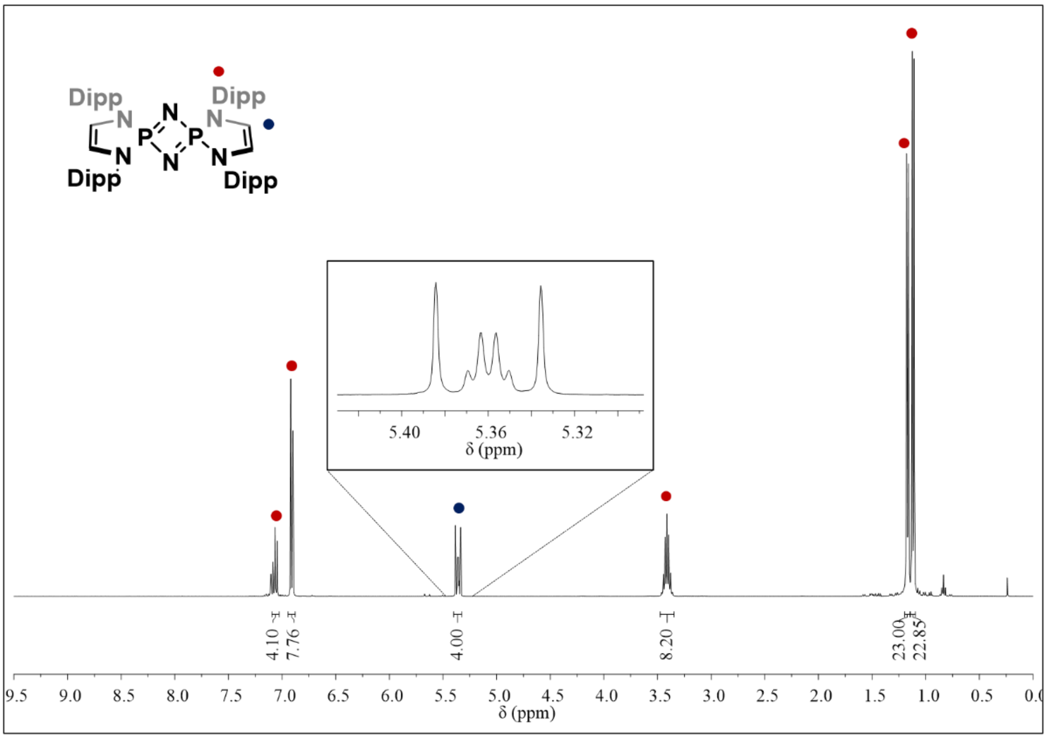

$^{13}C\{^{1}H\}$ NMR spectrum of **4** (100 MHz, C_6D_6)

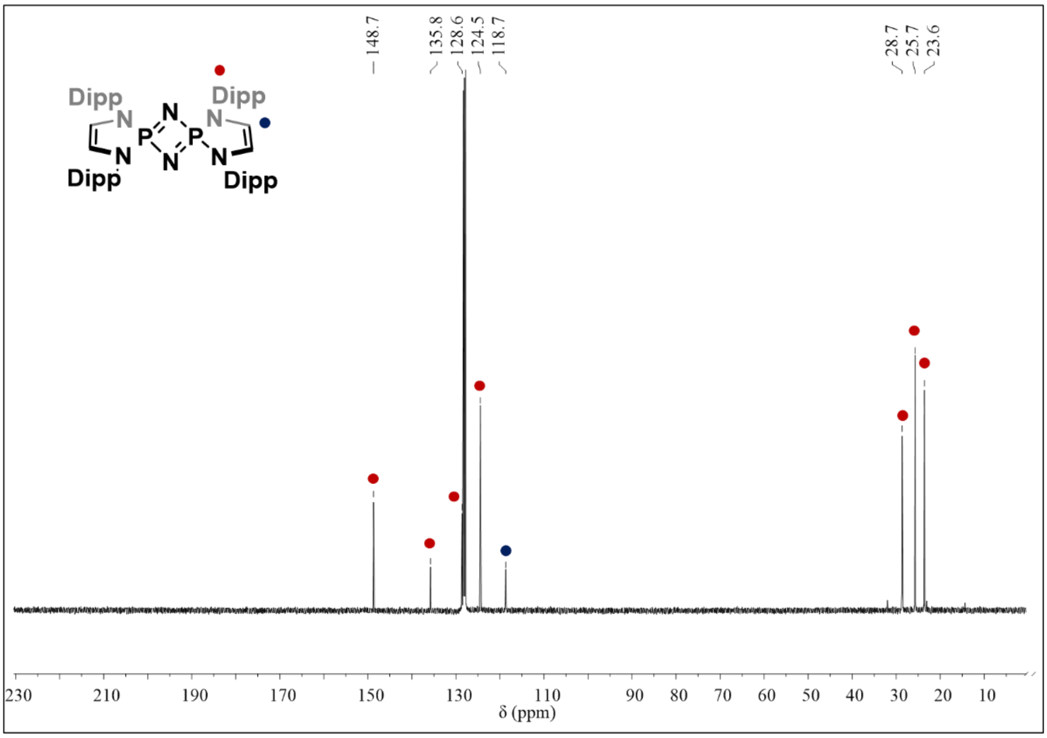

$^{31}P\{^{1}H\}$ NMR spectrum of **3** (162 MHz, C_6D_6)

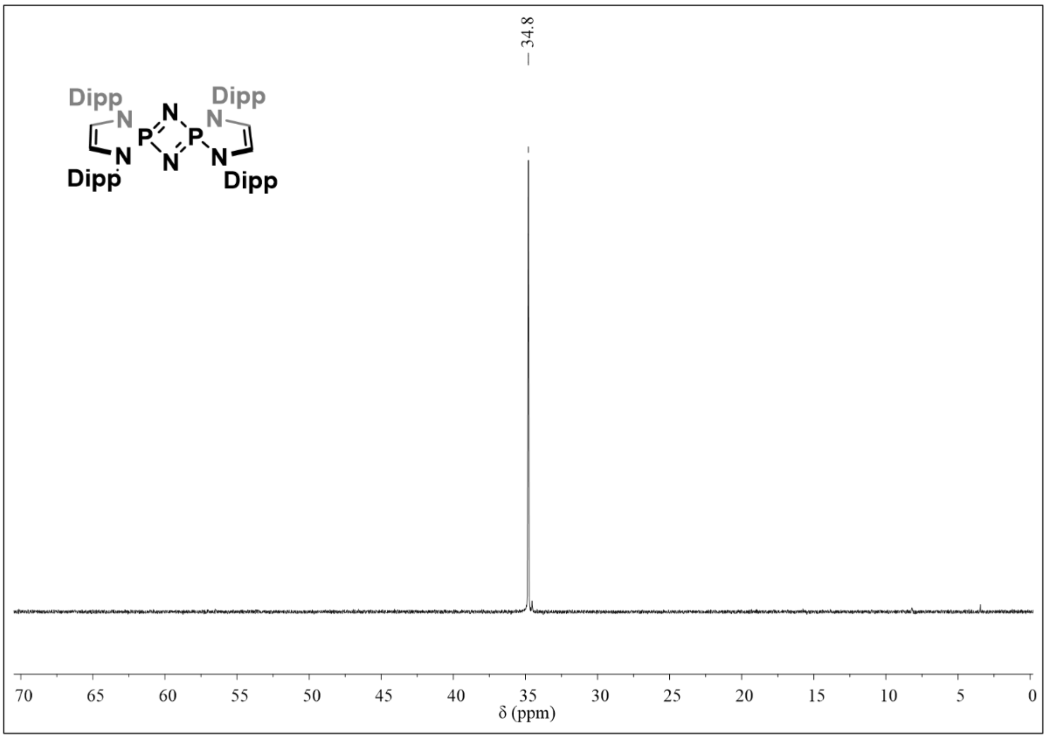

Supplement: Supplementary file 1 — ic4c04397_si_001.pdf [file ic4c04397_si_001.pdf]
